# Rare genetic variants in the *CFI* gene are associated with advanced age-related macular degeneration and commonly result in reduced serum factor I levels

**DOI:** 10.1093/hmg/ddv091

**Published:** 2015-03-18

**Authors:** David Kavanagh, Yi Yu, Elizabeth C. Schramm, Michael Triebwasser, Erin K. Wagner, Soumya Raychaudhuri, Mark J. Daly, John P. Atkinson, Johanna M. Seddon

**Affiliations:** 1 Institute of Genetic Medicine, Newcastle University, International Centre for Life, Newcastle upon Tyne, UK,; 2 Ophthalmic Epidemiology and Genetics Service, New England Eye Center, Tufts Medical Center, Boston, MA, USA,; 3 Division of Rheumatology, Department of Medicine, Washington University School of Medicine, St. Louis, MO, USA,; 4 Partners HealthCare Center for Personalized Genetic Medicine, Boston, MA, USA,; 5 Program in Medical and Population Genetics, Broad Institute, Cambridge, MA, USA,; 6 Division of Genetics, Rheumatology, Immunology and Allergy, Brigham and Women's Hospital, Boston, MA, USA,; 7 Faculty of Medical and Human Sciences, University of Manchester, Manchester, UK,; 8 Analytic and Translational Genetics Unit, Massachusetts General Hospital, Boston, MA, USA,; 9 Department of Ophthalmology, Tufts University School of Medicine, Boston, MA, USA and; 10 Sackler School of Graduate Biomedical Sciences, Tufts University, Boston, MA, USA

## Abstract

To assess a potential diagnostic and therapeutic biomarker for age-related macular degeneration (AMD), we sequenced the complement factor I gene (*CFI*) in 2266 individuals with AMD and 1400 without, identifying 231 individuals with rare genetic variants. We evaluated the functional impact by measuring circulating serum factor I (FI) protein levels in individuals with and without rare *CFI* variants. The burden of very rare (frequency <1/1000) variants in *CFI* was strongly associated with disease (*P* = 1.1 × 10^−8^). In addition, we examined eight coding variants with counts ≥5 and saw evidence for association with AMD in three variants. Individuals with advanced AMD carrying a rare *CFI* variant had lower mean FI compared with non-AMD subjects carrying a variant (*P* < 0.001). Further new evidence that FI levels drive AMD risk comes from analyses showing individuals with a *CFI* rare variant and low FI were more likely to have advanced AMD (*P* = 5.6 × 10^−5^). Controlling for covariates, low FI increased the risk of advanced AMD among those with a variant compared with individuals without advanced AMD with a rare *CFI* variant (OR 13.6, *P* = 1.6 × 10^−4^), and also compared with control individuals without a rare *CFI* variant (OR 19.0, *P* = 1.1 × 10^−5^). Thus, low FI levels are strongly associated with rare *CFI* variants and AMD. Enhancing FI activity may be therapeutic and measuring FI provides a screening tool for identifying patients who are most likely to benefit from complement inhibitory therapy.

## Introduction

Age-related macular degeneration (AMD) is a common disease with multifactorial etiology (1,2). In addition to behavioral and environmental factors, >20 common genetic loci have been confirmed to be associated with AMD, including many in the complement system (3–5). We have recently described strongly associated, rare, functionally significant variants in the complement factor H gene (*CFH*) (6,7), further suggesting that impaired complement regulation is central to the pathogenesis of AMD (7,8).

Recently, investigators have identified association of rare genetic variants in the complement factor I gene (*CFI*) with advanced AMD (AAMD) (9–11). These studies targeted *CFI* for sequencing because a common intronic variant in this gene was previously associated with risk of AAMD (12). The factor I (FI) protein is a serum serine protease that is a critical regulator of alternative pathway of complement activation by cleaving C3b in the presence of its cofactors (13,14).

The definition of the genetic factors driving AMD risk has led to the testing of novel therapeutic agents, including complement inhibitors. Results to date, however, have suggested that only a subgroup of individuals may respond to complement inhibition (15). That a disease with several disparate genetic predispositions should respond divergently to specific therapies should not be surprising. Personalized therapy for AMD based on an individual's genotype should be the ultimate goal; however, to date the majority of rare *CFI* genetic variants described in AMD patients are missense mutations of uncertain biologic relevance. Considering the loss-of-function *CFI* variant association with AMD, the potential of agents that prevent C3 activation (15) or supplement FI activity (16) to reduce the risk or progression of AMD and the need for better biomarkers, serum FI is a promising biomarker for disease diagnosis, prognosis and assessment of therapeutic efficacy.

We therefore interrogated consequences of these rare associated variants by studying FI antigenic levels in the circulation. We demonstrate that low serum FI levels are significantly associated with rare *CFI* variants and AAMD and suggest that this group of patients is most likely to benefit from complement inhibitory therapy (15) or supplementation with FI (16).

## Results

### *CFI* genetic analysis

**Figure 1.**
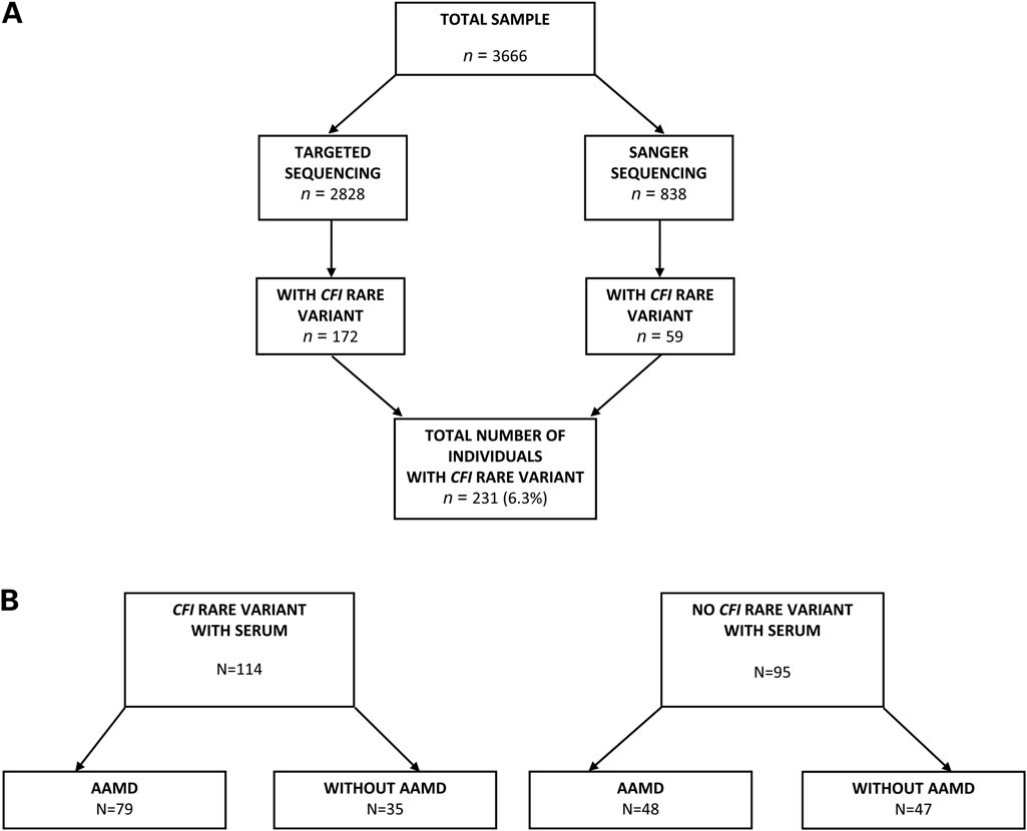
(**A**). Flow chart for sequencing and discovery of *CFI* rare variants. AAMD: advanced age-related macular degeneration. Targeted sequencing was performed on 2828 individuals, including 2421 previously sequenced unrelated AAMD cases and controls without any signs of AMD (145 individuals with *CFI* genetic variants) (9), and 407 additional unrelated cases and controls sequenced in the same manner (27 new individuals with *CFI* genetic variants identified). Sanger sequencing was performed on 838 individuals, including 396 cases with AAMD and 442 controls without any signs of AMD (59 new individuals with *CFI* genetic variants identified). A total of 231 subjects with *CFI* rare variants were identified. (**B**) Flow chart for selection of samples for serum analysis. In order to explore the effects of disease and *CFI* rare variants on serum FI levels, we first identified individuals with a variant and with AAMD for whom serum samples were available (*n* = 79). We identified three comparison groups from the sequenced individuals with similar mean ages at the time of blood draw compared with the advanced AMD cases carrying a variant: (i) subjects with AAMD but no variant (*n* = 48), (ii) a group without AAMD and without a variant (*n* = 47) and (iii) subjects without AAMD but with a variant (*n* = 35). The total sample comprised 209 subjects, with 114 carrying rare *CFI* variants.

In our study of 3666 AAMD and non-AAMD individuals (Fig. 1), 71 different non-synonymous *CFI* variants were detected among 231 individuals (Supplementary Material, Tables S1–S4), including ones classified as benign, possibly damaging, probably damaging, or loss-of-function by PolyPhen2. Variants classified as loss-of-function or probably damaging among variants with counts <5 in AAMD and non-AAMD (45 in AAMD versus 3 in non-AAMD) were significantly related to AMD (*P* = 6.1 × 10^−7^) (Supplementary Material, Table S4). Overall, there were far more rare, non-synonymous *CFI* variants in all categories combined in the AAMD group than the non-AAMD group (80 versus 10, *P* = 1.1 × 10^−8^) (Table 1; Supplementary Material, Table S4). Underscoring the matching of the case and control groups used in the sequencing analysis for ancestry and data quality, synonymous variants in *CFI* showed no difference (Table 1; Supplementary Material, Table S4).

**Table 1.** Pooled analysis for synonymous versus non-synonymous rare variants with counts <5

	Total AAMD	Total non-AMD	*P*-value
All non-synonymous variants	80	10	1.1 × 10^−8^
Synonymous variants	9	8	0.47

In addition, we examined the eight *CFI* variants with counts in AAMD or non-AAMD groups ≥5. No individual *CFI* genetic variant was highly associated with AAMD, although three were nominally associated: p.P553S (benign) with odds ratio (OR) of 2.69, *P* = 0.027; p.R406H (possibly damaging), OR 0.10, *P* = 0.015; and p.A240G (probably damaging), OR 7.43, *P* = 0.023 (Table 2; Supplementary Material, Tables S1–S3). This number of AAMD-associated variants (three of eight) is higher than would be expected by chance (*P* = 5.8 × 10^−3^).

**Table:** 2. Individual rare *CFI* variants with counts ≥5: association with AAMD and corresponding serum FI levels

Variant	OR (95% CI)	*P*-value	PolyPhen2 prediction	Mean serum FI (µg/ml)	Range serum FI (µg/ml)	% Low serum FI^a^
p.A240G^b^	7.43 (1.10–317.46)	0.02	Probably damaging	23.4	10.5–40.0	80 (4/5)
p.G119R	3.09 (0.68–14.13)	0.15	Probably damaging	26.1	22.0–28.1	100 (4/4)
p.P553S^b^	2.69 (1.11–6.54)	0.03	Benign	46.3	32.8–70.8	0 (0/9)
p.K441R	1.43 (0.74–2.75)	0.35	Benign	41.7	23.6–56.6	11 (2/18)
p.G261D	0.93 (0.38–2.27)	1.00	Benign	48.7	39.6–64.4	0 (0/12)
p.T300A	0.77 (0.21–2.88)	0.74	Benign	48.4	34.6–66.1	0 (0/7)
p.R202I	0.35 (0.11–1.10)	0.07	Possibly damaging	46.1^c^	35.1–62.6^c^	0 (0/11)^c^
p.R406H^d^	0.10 (0.01–0.85)	0.02	Possibly damaging	47.7	46.2–50.5	0 (0/3)

AAMD, advanced age-related macular degeneration; *CFI*, complement factor I gene; FI, serum factor I.

^a^Percentage (and number) of subjects with the variant who have low serum FI levels (<29.3 µg/ml).

^b^Risk variant.

^c^One individual was a compound heterozygote (p.R202I/p.A356P). This individual had a normal serum FI level (35.6 µg/ml) and is not shown in this table.

^d^Protective variant.

Of the 209 individuals who had serum analyses, 114 subjects had a rare genetic variant in *CFI*, of which 79 (69%) had AAMD. These rare genetic variants occurred throughout the gene with amino acid changes in both heavy and light chains of FI and with no obvious mutational hot spots (Supplementary Material, Fig. S1 and Tables S1, S2).

### Analysis of *CFI* rare variant carriers

Functional consequences of these rare variants were assessed by evaluating serum FI antigenic measurements. Table 2 displays the FI serum levels for eight individual *CFI* variants analyzed for association with AAMD. Among the five individuals with the p.A240G risk variant and classified as probably damaging (all with AAMD), four of the five had low serum FI levels. All individuals carrying the p.P553S risk variant and classified as benign (eight AAMD, one non-AAMD) had normal FI levels. Among the three individuals with the protective p.R406H variant classified as possibly damaging (one AAMD, two non-AAMD), the serum FI levels were normal. The previously reported p.G119R risk variant (9,11) did not formally reach statistical significance in our study; however, all four carriers of this variant were AAMD cases and had low FI levels, consistent with an association.

**Figure 2.**
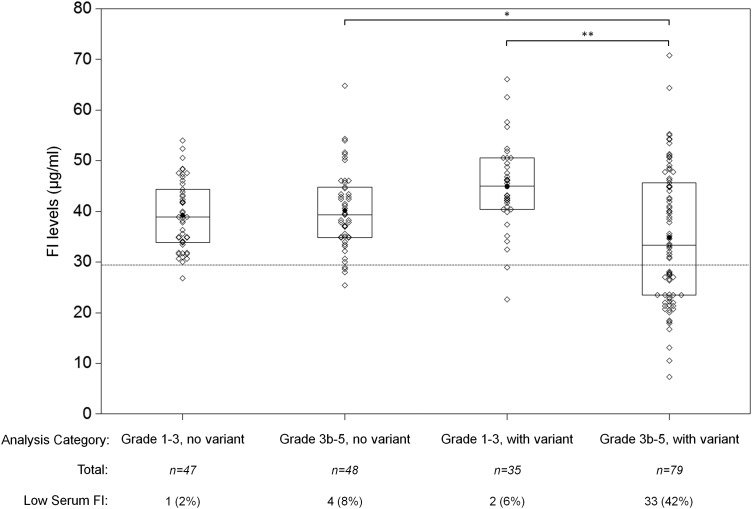
Comparison of serum FI levels in advanced age-related macular degeneration (AAMD) and non-AAMD/*CFI* rare variant groups. Serum FI levels were measured for 209 individuals. The mean of the 79 patients with AAMD and rare genetic *CFI* variants was 34.8 μg/ml (95% CI: 31.8–37.8). For the 48 AAMD patients with no rare genetic *CFI* variants, the mean was 40.1 μg/ml (95% CI: 37.8–42.4). In those without AAMD and with genetic variants (*n* = 35), the mean was 44.8 μg/ml (95% CI: 41.8–47.9). In the non-AAMD without a genetic variant (*n* = 47) the mean was 39.2 μg/ml (95% CI: 37.2–41.2). The box plots show medians and quartiles. The means are shown as black circles. Individual patient values are shown as diamonds. Analysis of variance was undertaken by one-way ANOVA with *post hoc* analysis. The lower limit of normal is demonstrated by a dotted line (29.3 μg/ml) (*P = 0.03, **P < 0.001). Subjects with low serum FI levels are shown as *n* (%).

As shown in Figure 2, the average serum FI level in those with AAMD and a *CFI* variant was significantly lower than among those without AAMD and a *CFI* variant (34.8 versus 44.9 µg/ml, *P* < 0.001). Among individuals without a *CFI* variant, there was no significant difference between AAMD (40.1 µg/ml) and the comparison group (39.2 µg/ml). Among all individuals with AAMD, the mean FI level was significantly lower among those carrying a rare genetic variant (*P* = 0.03). A larger percentage of individuals with AAMD and a rare *CFI* variant had low serum FI, compared with the comparison groups: AAMD without a variant, non-AAMD without a variant, and non-AAMD with a variant (*P* < 0.001). Therefore, rare *CFI* variants are lowering serum FI levels and contributing to AMD risk, whereas serum FI is not contributing to AMD risk among individuals without a variant.

**Figure 3.**
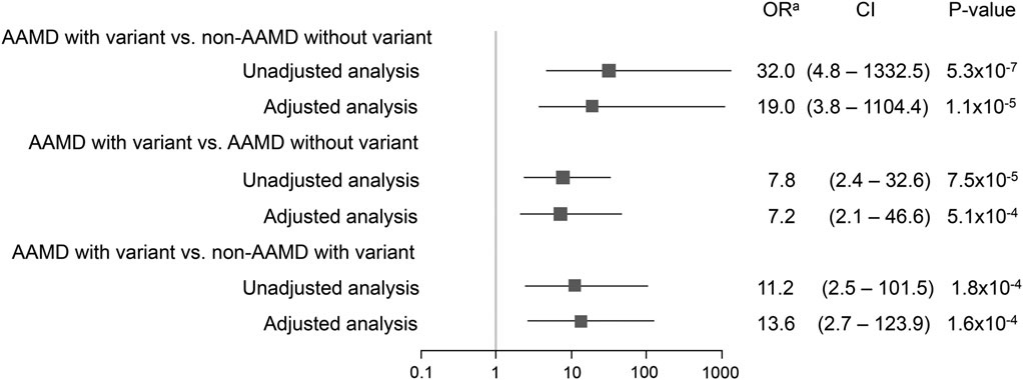
The association between groups defined by advanced age-related macular degeneration (AAMD)/*CFI* rare variant status and low serum FI level. Unadjusted analyses: estimated odds ratio of the association between AAMD/*CFI* rare variant status and low serum FI (≤29.3 µg/ml). Adjusted analyses: estimated odds ratio of the association between AAMD/*CFI* rare variant status and low serum FI (≤29.3 µg/ml) after adjusting for age at the time of blood sample (≤80, >80 yr), gender, body mass index (<25, ≥25) and smoking status (never, ever). All analyses used using exact binomial probabilities based on the exact option of PROC FREQ in SAS 9.3. OR: odds ratio; CI: confidence interval. ^a^OR > 1 indicates that low serum FI increases risk, while OR < 1 indicates that low serum FI is protective.

Figure 3 shows the estimated ORs for the associations among three comparison groups defined by AAMD, rare *CFI* variant status and low serum FI levels. Without adjustment for covariates, having low serum FI strongly increased risk of AAMD among those with a variant (OR 32.0, *P* = 5.3 × 10^−7^) compared with individuals without AAMD and without a variant. After adjustment for age, gender, body mass index (BMI) and smoking, the OR was 19.0, *P* = 1.1 × 10^−5^. When comparing AAMD with a variant to AAMD without a variant, the OR's for low serum FI were 7.8 in the unadjusted model (*P* = 7.5 × 10^−5^) and 7.2 (*P* = 5.1 × 10^−4^) for the adjusted model. For analyses only among individuals with a rare variant, low serum FI was associated with AAMD with OR 11.2 in the unadjusted model (*P* = 1.8 × 10^−4^) and 13.6 (*P* = 1.6 × 10^−4^) in the adjusted model. Thus, low serum FI is a risk factor for AAMD in association with a rare *CFI* variant.

Table 3 displays the distribution of variables according to serum FI level among all subjects with a rare variant. Individuals with low serum FI were more likely to have AAMD (94.3 versus 58.2%, *P* = 5.6 × 10^−5^). Males tended to have lower FI levels (*P* = 0.02) and there was no association with age (*P* = 0.68). There was also no association with smoking or BMI (data not shown).

**Table 3.** Distribution of variables according to serum FI level among all subjects with a rare *CFI* variant

Variable	Low serum FI (*n* = 35)	Normal/high serum FI (*n* = 79)	*P*-value^a^
Age at sample (mean ± SD)	76.6 ± 9.5	75.9 ± 8.1	0.68
Gender (*n*, % male)	19 (54.3)	24 (30.4)	0.02
AMD groups^b^			
Non-AAMD	2 (5.7)^c^	33 (41.8)	5.6 × 10^−5^
AAMD	33 (94.3)	46 (58.2)	

*CFI*, complement factor I (gene); FI, factor I (protein).

Variables are presented as *n* (%) or (mean ± SD).

^a^*P*-values calculated as follows: one-way ANOVA for age at sample; Fisher's exact test for AMD Groups.

^b^AMD classifications: non-AAMD: no AMD, early or intermediate AMD defined as subjects with CARMS grades (17), 2 or 3a in at least one eye and no advanced disease in the worst eye; AAMD, advanced AMD defined as subjects with CARMS grades 3b, 4 or 5 in at least one eye.

^c^For the two individuals in the non-AAMD group with a type 1 mutation, retinal screening at the beginning of the study revealed grade 3 maculopathy at ages 42 and 73, respectively. With additional follow-up time, the 73-year-old individual has progressed to grade 4 (advanced dry AMD).

**Figure 4.**
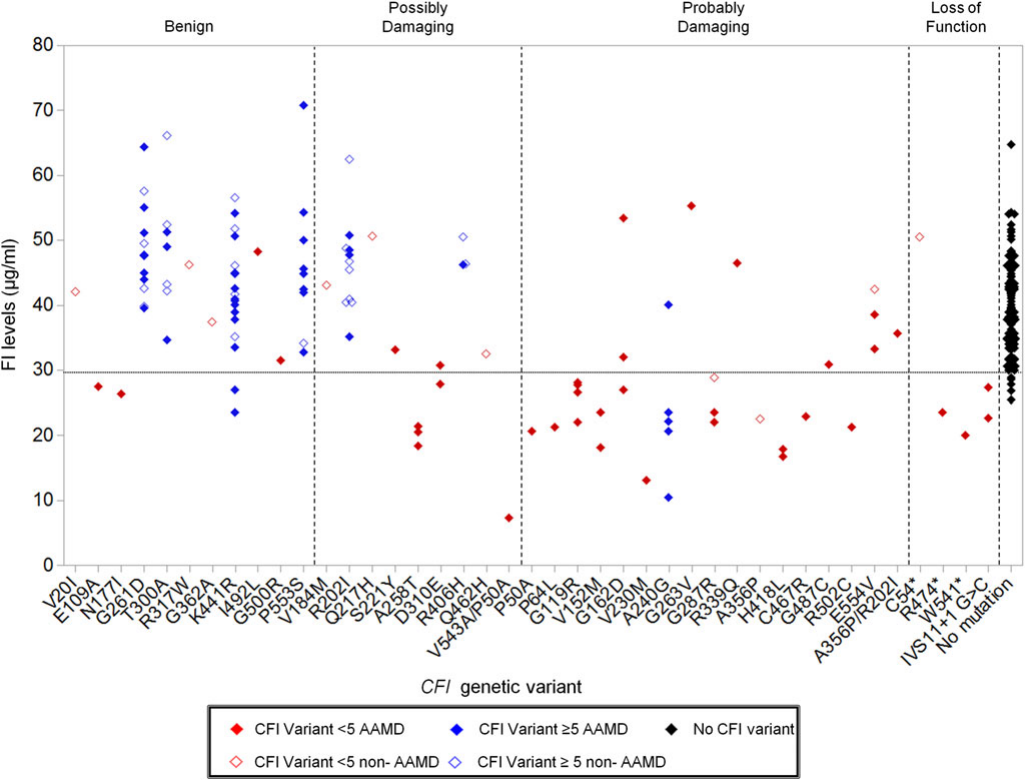
Serum FI levels by rare *CFI* genetic variant. The serum FI levels of all individuals stratified by *CFI* genetic variant. *CFI* variants with counts <5 in the combined sequencing panel are colored red. Variants with counts ≥5 are blue. Genetic variants in individuals with AAMD are represented by a filled diamond and comparison group without AAMD are shown with an unfilled diamond. Genetic variants are grouped into benign, possibly damaging, probably damaging, or loss of function as predicted by PolyPhen2. Two individuals were compound heterozygotes (p.V543A/p.P50A; p.A356P/p.R202I). p.R202I and p.V543A were predicted to be possibly damaging while A356P and P50A were predicted to be probably damaging. The black filled diamonds represent the 95 individuals (47 non-AAMD, 48 AAMD) with no *CFI* variants. The lower limit of normal is demonstrated by a dotted line (29.3 μg/ml).

As shown in Figure 4, among the 79 individuals with AAMD and a *CFI* variant, 33 had low FI levels. On the other hand, among the 35 subjects without AAMD and a *CFI* variant, only 2 had low FI levels. Twenty-one different genetic variants were seen in association with low FI levels.

**Figure 5.**
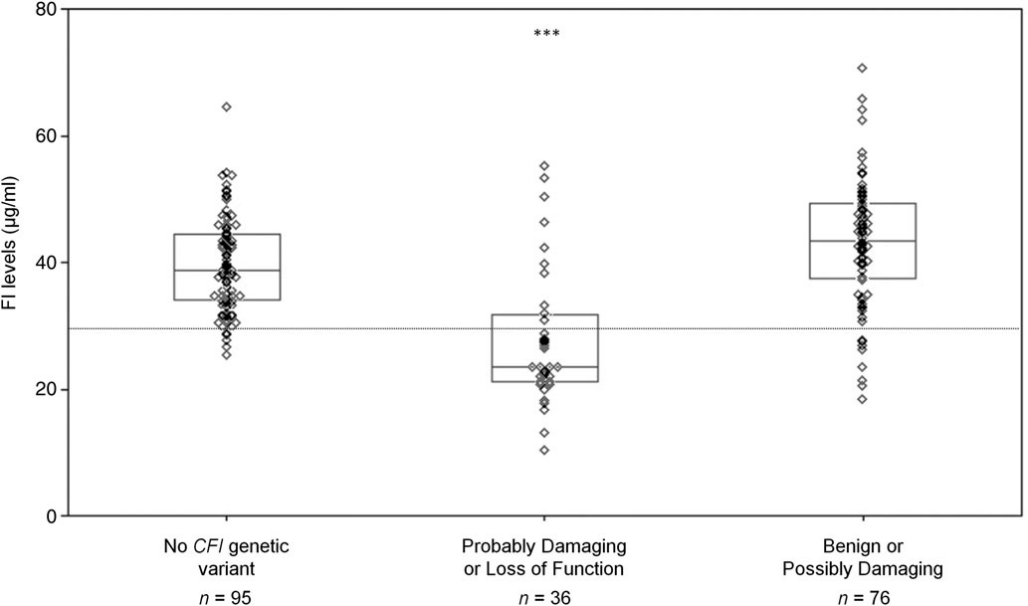
Comparison of serum FI levels by *in silico* functional prediction. The mean of the 76 individuals with rare genetic *CFI* variants predicted to be either benign or possibly damaging was 43.1 μg/ml (95% CI: 40.8–45.5). The 36 individuals with probably damaging or loss-of-function *CFI* variants had a mean serum FI level of 27.7 μg/ml (95% CI: 24.0–31.4). In those with no rare genetic variants in *CFI* (*n* = 95), the mean was 39.7 μg/ml (95% CI: 38.2–41.2). The box plots show medians and quartiles. The means are shown as dark circle. Individual patient values are shown as diamonds. The two individuals carrying compound heterozygous mutations were not included in this analysis. Analysis of variance was undertaken by one-way ANOVA with *post hoc* analysis (***P < 0.001). The lower limit of normal is demonstrated by a dotted line (29.3 μg/ml).

Figure 5 demonstrates the serum FI levels categorized by the *in silico* prediction of functional consequence of the genetic variant. FI levels were significantly lower in individuals with probably damaging or loss-of-function *CFI* variants compared with those with benign or loss-of-function variants (mean 27.7 versus 43.1 µg/ml, *P* < 0.001) as well as compared with those with no *CFI* variant (27.7 versus 39.7 µg/ml, *P* < 0.001).

### Analyses of common *CFI* variant and serum C3

In the 95 individuals with no rare genetic variants in *CFI*, FI levels were assessed in relation to the common variant associated with AMD, rs10033900, near the *CFI* gene (12). The mean FI level for the 21 individuals homozygous for the C allele was 38.7 μg/ml and for the 25 individuals homozygous for the risk allele (T) it was 38.9 μg/ml (Supplementary Material, Fig. S2), indicating no significant difference in FI levels related to the common *CFI* variant.

C3 levels were measured in 32 individuals with AAMD and a *CFI* variant associated with low FI levels (type 1 *CFI* mutation) and 13 individuals with grade 1 only and no *CFI* variants. Only three individuals with a type 1 *CFI* mutation had low C3 levels (Supplementary Material, Fig. S3). There was a positive correlation between the levels of FI and C3 in these 32 individuals with a type 1 *CFI* variant (*R* = 0.51, *P* = 0.003) (Supplementary Material, Fig. S4).

## Discussion

In this study, we demonstrated that individuals with AAMD and rare *CFI* variants commonly have low serum FI antigenic levels. This result, observed in association with 21 variants, is highly suggestive of a variant causing a structural effect on the mutant protein, impairing secretion. Further, this provides compelling data corroborating previous genetic evidence for the role of impaired FI-mediated regulation of the complement system's alternative pathway in the pathogenesis of AMD (9–11). Among all individuals with a rare *CFI* genetic variant, those with AAMD were significantly more likely to have low FI levels (haploinsufficiency) compared with those without AAMD (13.6-fold higher). Type 1 *CFI* mutations accounted for 42% of the AAMD group but only 6% of the non- AAMD group.

In addition to its role in the pathogenesis of AMD, partial deficiency (haploinsufficiency) of FI is associated with atypical Hemolytic Uremic Syndrome (aHUS: MIM 612923) (18). FI is one of a number of biomarkers used in aHUS to discriminate between complement-mediated aHUS (e.g. *CFI*, *CFH* and *CD46* genes) which will respond to the complement inhibitor, Eculizumab and the non-complement-mediated forms (e.g. *DGKE* gene) that do not (19).

Twenty-one distinct genetic variants were identified in association with low FI levels in the AMD population. Of these variants, 10 have been seen in aHUS patients associated with low levels (18) (p.P50A (20–22), p.P64L (20), p.G119R (20,21,23), p.V152M (24),p.G162D (25,26), p.N177I (27), p.A240G (22,28,29), p.G287R (23), p.K441R (30), p.R474 (21,22,24,31)).

Among rare variants with counts ≥5, the p.A240G variant nominally increased risk of AMD in our study. Among the five individuals with this variant and serum FI levels, four had low levels ranging from 10.5 to 23.5 and one had a normal value. The previously reported p.G119R risk variant (9,11) was not significant in our study (*P* = 0.15) but had low FI in all individuals tested (Table 2 and Fig. 4).

There were no confirmed cases of aHUS in our cohort of AMD patients with *CFI* variants. It is believed that a confluence of rare genetic variants and common genetic polymorphisms and haplotypes in addition to a triggering stimulus, such as infection or pregnancy, are required for aHUS to manifest (18). It is known that different *CFH* risk haplotypes exist for aHUS and AMD (32). It is possible that differences in the background complotype (33) of an individual may determine the ultimate clinical disease process. Further investigation is required to fully understand the genetic differences between *CFI*-mediated aHUS and AMD.

Individuals carrying one of four variants, p.G162D, p.A240G, p.D310E, and p.K441R, had both normal and low FI levels. FI is an acute phase protein with IL6 (34) and IFNγ (35,36) increasing its secretion. In those individuals with no genetic variants, we demonstrated up to an approximate 2-fold variation in levels in keeping with previous studies (Fig. 2) (37). With such natural variability, it is possible that the effects of a heterozygous variant may be masked in some patients by the wide normal range, thus requiring care when interpreting serum levels in isolation. However, a value <29.3 µg/ml is a strong indicator of risk for AMD.

While the functional effect of type 1 *CFI* genetic variants in the pathogenesis of AMD seems apparent, the effect of the rare genetic variants which do not result in a failure of secretion (type 2 variant) remains unclear. Of the type 2 variants, p.P553S, classified as benign by PolyPhen2, increased risk for AMD (OR 2.7), while p.R406H classified as possibly damaging, was protective (OR 0.10). Functional analyses of the type 2 variants p.G261D and p.R406H have not shown a loss of activity (38,39), while further analysis of the remaining variant proteins with serum antigenic levels in the normal range will be required to assess their role in disease. We are taking two approaches to address this question: expression of the mutant in question and development of a test employing an individual's serum that closely correlates with antigenic levels.

There was no significant difference in FI levels in AAMD and non-AAMD individuals without a *CFI* genetic variant in keeping with a causative role for the genetic variants. In those individuals without a rare genetic variant, we also assessed FI levels in the context of a common *CFI* variant previously associated with AMD (rs10033900) (12,40). We did not demonstrate significant differences in FI levels by rs10033900 genotype.

FI is required to downregulate the alternative pathway of complement activation and complete FI deficiency is associated with a consumptive deficiency of C3 and consequently recurrent infections, not immunopathology (13,14). It is informative that in our study no individual with AMD was seen to have complete deficiency of FI. One individual with two genetic variants (P50A/V543A), who we assume to be a compound heterozygote, had very low, although detectable, FI levels (7.3 µg/ml). Interestingly, this patient's C3 level was normal (0.94 mg/ml), and it can be assumed that even in this individual there is sufficient functional FI present. In the complete absence of FI, no efficient breakdown of C3b to iC3b and ultimately C3c and C3dg is possible. The formation of iC3b and its interaction with CR3 and CR4 is one of the most potent complement inflammatory effects (14). In a *CFI* knockout mouse, C3 circulates as C3b with no evidence of further degradation products (e.g. iC3b, C3dg) (41). This mimics the situation in humans with complete deficiency of FI. These C3b metabolites have been demonstrated to be critical in the development of membranoproliferative glomerulonephritis in a mouse model of disease (41,42). Our study is in keeping with a hyperinflammatory complement phenotype causing AMD at least in part mediated through iC3b (14).

Our hypothesis to explain the AMD/FI association is straightforward: increased complement alternative pathway activity in the retina results from or is secondary to low FI levels; i.e. for a given degree of retinal injury or damage, there is an excessive (undesirable) degree of complement activation which is particularly damaging to the retina. The hyperinflammatory response may affect the retinal pigment epithelium, drusen, or the local vasculature, leading to an acceleration of the disease process. This conforms to established complement risk factors for AMD which can cause an accelerated C3b feedback cycle. Unsurprisingly, it has long been known that elevating serum FI concentration can downregulate this C3b amplification loop (43).

It is interesting to note, although entirely in keeping with *CFI*-associated aHUS (18), that even in AAMD cases with type I *CFI* mutations, 29 of 32 had normal C3 levels. C3 is also an acute phase protein and not a sensitive marker of complement activation. In these patients, however, there was a positive correlation between FI levels and C3 levels in keeping with the regulatory activity of FI.

Rare genetic variants in *CFI* have been associated with AAMD (9–11); however, a majority are missense mutations of uncertain clinical relevance. This inability to correctly identify functionally significant genetic variants impairs our understanding of the disease pathogenesis and is an impediment to personalized management of AMD. Our data underscore the pathogenic role of *CFI* genetic variants in AMD. Measuring serum antigenic levels of FI provides a practical, low cost, screening tool for AMD in the clinic. We suggest that this group of patients with both a rare *CFI* variant and low serum FI is most likely to benefit from complement inhibitory therapy (15) or supplementation with FI (16). We note that the recent MAHALO study examining the role of lampalizumab (anti-factor D complement regulator) in AMD reported success in a subgroup of patients with the common *CFI* variant (15). Identification of individuals with type I *CFI* mutations will allow targeting of those most likely to benefit from complement inhibitory therapy and pave the way for personalized medicine in AMD (44).

## Materials and Methods

### Study population

All individuals in this study were Caucasian participants in ongoing genetic and epidemiologic studies of macular degeneration who signed consent forms for the study approved by the institutional review board (6,7,10,17,44–50). We determined presence or absence of AMD based on clinical examination and fundus photography. JMS assigned grades for no AMD, early, intermediate and advanced stages of AMD using the Clinical Age-Related Maculopathy Grading System (CARMS) (17) as follows: Grade 1-no drusen or only a few small drusen; Grade 2-early maculopathy with small, hard drusen (2a) or retinal pigment alteration (2b) or both (2c); Grade 3-intermediate AMD with several intermediate drusen and/or presence of large, soft drusen; and AAMD with Grade 3b-drusenoid retinal pigment epithelial detachment, Grade 4-central or non-central geographic atrophy, and Grade 5-neovascular AMD.

### Serum samples and complement analysis

We obtained serum samples according to a standard protocol and stored them at −140^°^C (51). We measured FI levels by radial immunodiffusion with anti-human antibodies specific for FI in 1% agarose gels (normal range 29.3–58.5 µg/ml) (51,52). C3 was measured by standard clinical laboratory nephelometric methods (normal range 0.79–1.67 mg/ml) (51).

### Sequencing the *CFI* gene

Figure 1 displays the source of all sample subsets described in this manuscript. To elaborate, we previously reported a targeted sequencing study of samples from 1676 individuals with AAMD and 745 subjects without any signs of AMD, covering 681 genes including *CFI* (10). We expanded this cohort by sequencing additional independent samples of 194 cases and 213 controls using the same targeted sequencing design (10). In that cohort of total 2828 samples, we identified 172 individuals who had at least one rare allele in *CFI*. We further extended this search for variants by conducting Sanger sequencing of the *CFI* gene in a separate and independent cohort with a total sample size of 838 (396 persons with AAMD and 442 without any signs of AMD with grade 1 in both eyes or grade 1 in one eye and grade 2 in the fellow eye). Using the same primers previously shown to produce high quality amplicons covering the entire exon sequence and adjacent splice sites (10), we sequenced forward and reverse PCR primers via capillary electrophoresis employing ABI Prism 3730xl DNA analyzers (Applied Biosystems, Foster City, CA, USA) at Beckman Coulter Genomics. Chromatograms were assembled using CONSED and polymorphisms were detected using polyphred 5.04 (University of Washington), with proprietary improvements to increase sensitivity while maintaining specificity. We identified an additional 59 individuals in the Sanger sequencing study who had a least one rare allele in *CFI*. (Supplementary Material, Tables S1–S4 show the results of the combined sequencing experiments).

### Serum sample selection

In order to explore the effects of disease and *CFI* rare variants on serum FI levels, we first identified individuals with a variant and with AAMD for whom serum samples were available (*n* = 79). We identified three comparison groups from the sequenced individuals with similar mean ages at the time of blood draw compared to advanced AMD cases carrying a variant: (i) subjects with AAMD but no variant (*n* = 48); (ii) subjects without AAMD and without a variant (*n* = 47); and (iii) subjects without AAMD but with a variant (*n* = 35) (Fig. 2). The total sample comprised 209 subjects, with 114 carrying rare *CFI* variants.

### Statistical analysis

We obtained information on covariates including age at blood sample collection, gender, BMI and smoking from our AMD study databases as previously described (6,10,17,44–50). We coded BMI and smoking as dichotomous variables: BMI (<25, ≥25), smoking (never smoked, ever smoked). Serum FI status was coded as a dichotomous variable according to laboratory normal ranges (51), with serum FI under 29.3 µg/ml being classified as low serum FI level. The relationship between age at blood sample collection and serum FI level with AMD/*CFI* rare variant status was assessed using a one-way ANOVA. Pearson's Chi-square test was used for gender, BMI, smoking status and low serum FI level.

To calculate the OR of the association between AAMD/*CFI* rare variant status and low serum FI, we classified individuals according to their age (<80 yrs or 80+ yrs), gender, BMI and smoking status. The OR for non-adjusted and covariate adjusted associations were computed using exact binomial probabilities by PROC FREQ procedure with EXACT option of SAS 9.3.

For analyses of subjects with a rare *CFI* genetic variant, we used binary logistic regression analysis to analyze the association between a subject's AAMD status and serum FI, controlling for age at blood sample collection, gender, smoking and BMI status. Subjects with AAMD were compared to individuals with a rare *CFI* variant but without AAMD. For the rare *CFI* variants (defined as a frequency <1% in either the AAMD or non-AAMD groups), we tested variants with a frequency ≥5 among AAMD and non- AAMD groups separately. For variants with frequency <5, we grouped variants into categories based on PolyPhen2 (53), and analyzed them for significance within each group. We estimated allele frequencies of *CFI* variants using PLINK (54), and evaluated association of single variant or a group of variants with AAMD by Fisher's exact test.

## Supplementary Material

Supplementary Material is available at *HMG* online.

## Funding

This work was supported in part by National Institute of Health grants R01-EY11309 (J.M.S.), K08AR055688 (S.R.), U01HG0070033 (S.R.), F30HL103072 (M.T.), R01-AI041592 (J.P.A.), U54 HL112303 (J.P.A.); Edward N. & Della L. Thome Memorial Foundation (J.P.A.); the Doris Duke Clinical Scientist Development Award; the Protein Core Facility of the Rheumatic Diseases Core supported by National Institute of Arthritis and Musculoskeletal and Skin Diseases, part of the National Institutes of Health, under award number P30 AR48335 (J.P.A.), Massachusetts Lions Eye Research Fund, Inc. (J.M.S.); the Foundation Fighting Blindness (J.M.S.); Research to Prevent Blindness Challenge grant to the New England Eye Center, Department of Ophthalmology, Tufts University School of Medicine; American Macular Degeneration Foundation (J.M.S.); and the Macular Degeneration Research Fund of the Ophthalmic Epidemiology and Genetics Service, New England Eye Center, Tufts Medical Center, Tufts University School of Medicine. D.K. is a Wellcome Trust Intermediate Clinical Fellow and is funded by Fight for Sight. The content is solely the responsibility of the authors and does not necessarily represent the official views of the National Institutes of Health. Funding to pay the Open Access publication charges for this article was provided by the Wellcome Trust.

*Conflict of Interest statement*. None declared.

## Supplementary Material

Supplementary Data
