# Supplementary material for: Rare genetic variants in the CFI gene are associated with advanced age-related macular degeneration and commonly result in reduced serum factor I levels
Source: Hum Mol Genet. 2015 Mar 18;24(13):3861–70. doi: 10.1093/hmg/ddv091 (PMC4459386; doi:10.1093/hmg/ddv091)
Supplement: Supplementary Data [file supp_24_13_3861__index.html]

Rare genetic variants in the CFI gene are associated with advanced age-related macular degeneration and commonly result in reduced serum factor I levels — Rare genetic variants in the CFI gene are associated with advanced age-related macular degeneration and commonly result in reduced serum factor I levels — Supplementary Data 

# Rare genetic variants in the *CFI* gene are associated with advanced age-related macular degeneration and commonly result in reduced serum factor I levels

## Supplementary Data

Supplementary Data

**Files in this Data Supplement:**

- Supplementary Figures - doc file
- Supplementary Tables - doc file
