# Supplementary material for: Rare genetic variants in the CFI gene are associated with advanced age-related macular degeneration and commonly result in reduced serum factor I levels
Source: Hum Mol Genet. 2015 Mar 18;24(13):3861–70. doi: 10.1093/hmg/ddv091 (PMC4459386; doi:10.1093/hmg/ddv091)
Supplement: Supplementary Data [file supp_ddv091_ddv091supp_figs.doc]

**SUPPLEMENTARY FIGURES**

**
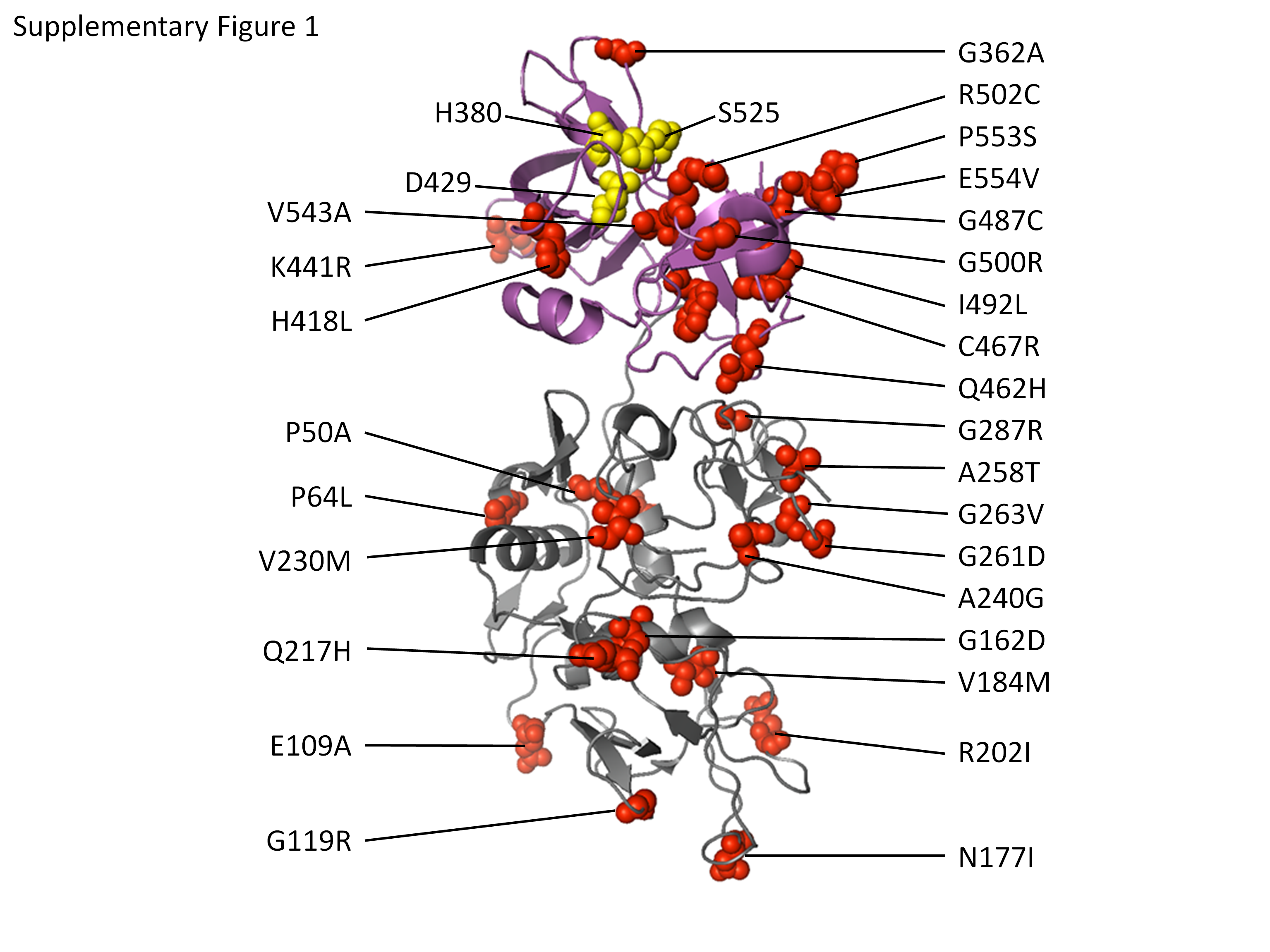
**

**Supplementary Figure 1.** Location of rare *CFI* genetic variants within the crystal structure of complement factor I. FI is a heterodimer consisting of a non-catalytic heavy chain (grey) linked by a disulfide bond to a catalytic light chain (magenta). Genetic variants described in this study with FI levels available are shown as red spheres. The variants: p.P64L; p.E109A; p.G119R; p.N177I; p.R202I; p.Q217H; p.V230M; p.A258T; p.G261D; p.G263V; p.T300A; p.A356P; p.G362A; p.K441R; p.Q462H; p.G487C; p.I492L; p.G500R; p.R502C; p.P553S; and p.E554V map to surface regions of the protein. Yellow spheres mark the catalytic triad of the serine protease domain. The protein database identification code for which this graphic was constructed is 2XRC.

**
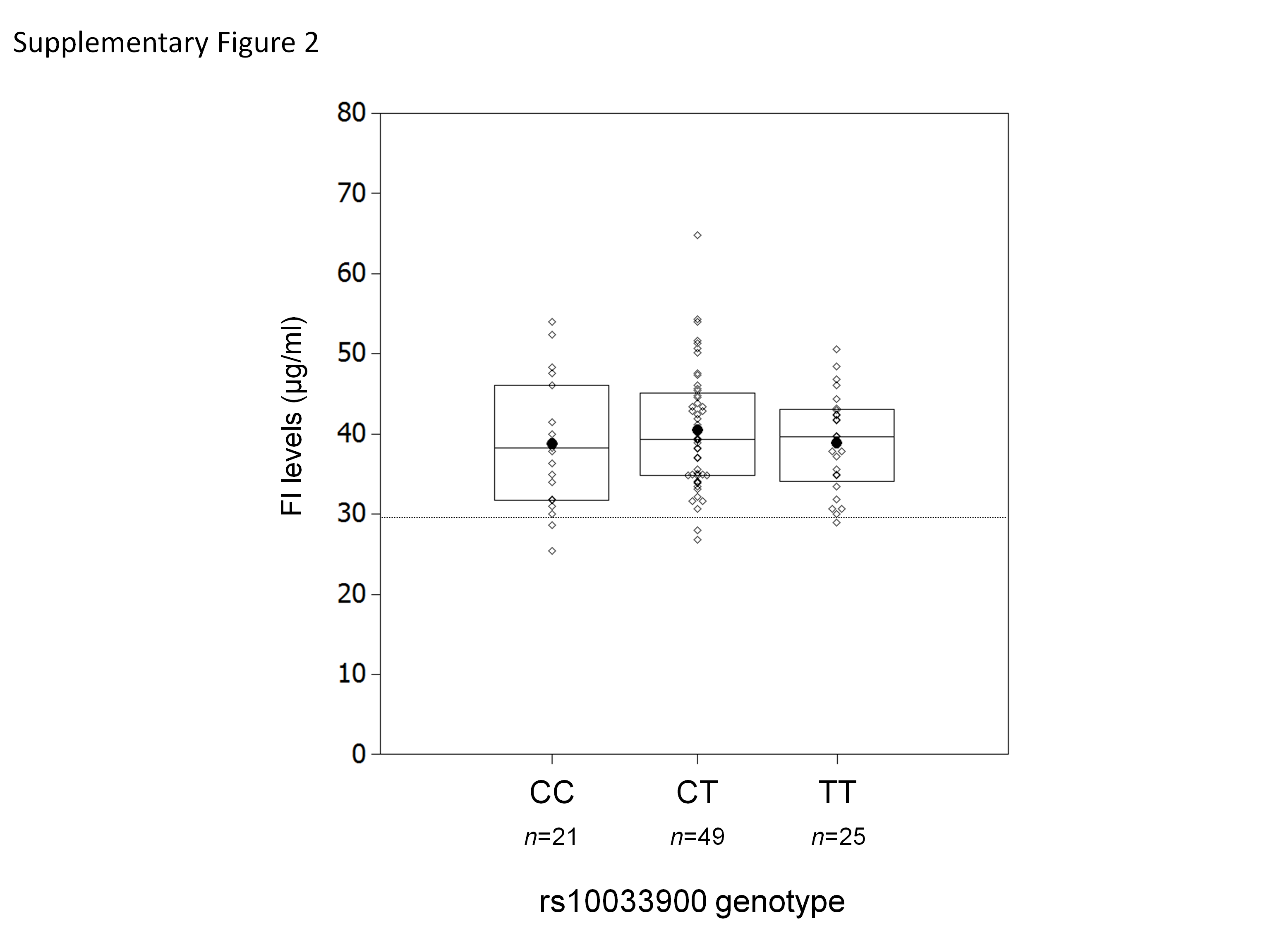
**

**Supplementary Figure 2.** Effect of common *CFI* variant (rs10033900), on plasma complement FI level among 95 individuals with no *CFI* variants. The means are shown as black circles (CC 38.7 μg/ml, (95% CI: 35.0 - 42.4); CT 40.4 μg/ml (95% CI: 38.2 - 42.6); TT 38.9 μg/ml (95% CI: 36.4 - 41.4)). The lower limit of normal is demonstrated by a dotted line (29.3 μg/ml). Analysis of variance was undertaken by one-way ANOVA (Minitab16) and there was no significant difference. The box plots show medians and quartiles. Individual patient values are shown as diamonds.

**
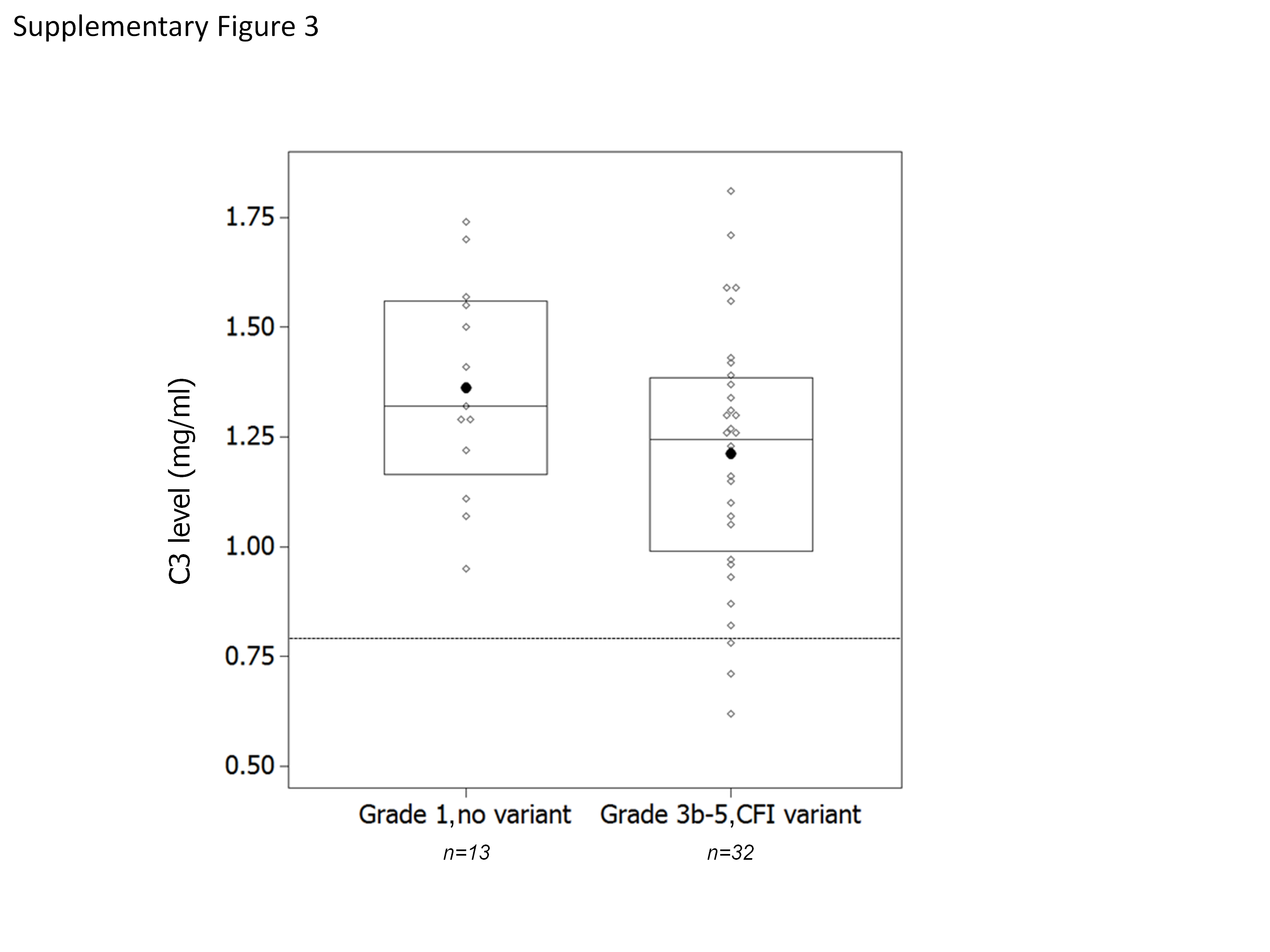
**

**Supplementary Figure 3** Serum C3 levels by AMD grade. The C3 levels were measured in 32 patients with AAMD and a rare genetic variant associated with a low FI serum level. C3 levels were also measured in 13 individuals without any signs of AMD (grade 1) and no *CFI* variants. The mean C3 level in the non-AAMD population is greater than in the AAMD patients (1.36 mg/ml vs. 1.21 mg/ml, P = 0.04, 2-sample t-test). The lower limit of normal is demonstrated by a dotted line (0.79 mg/ml). The box plots show medians and quartiles. The means are shown as black circles. Individual values are shown as diamonds.

**
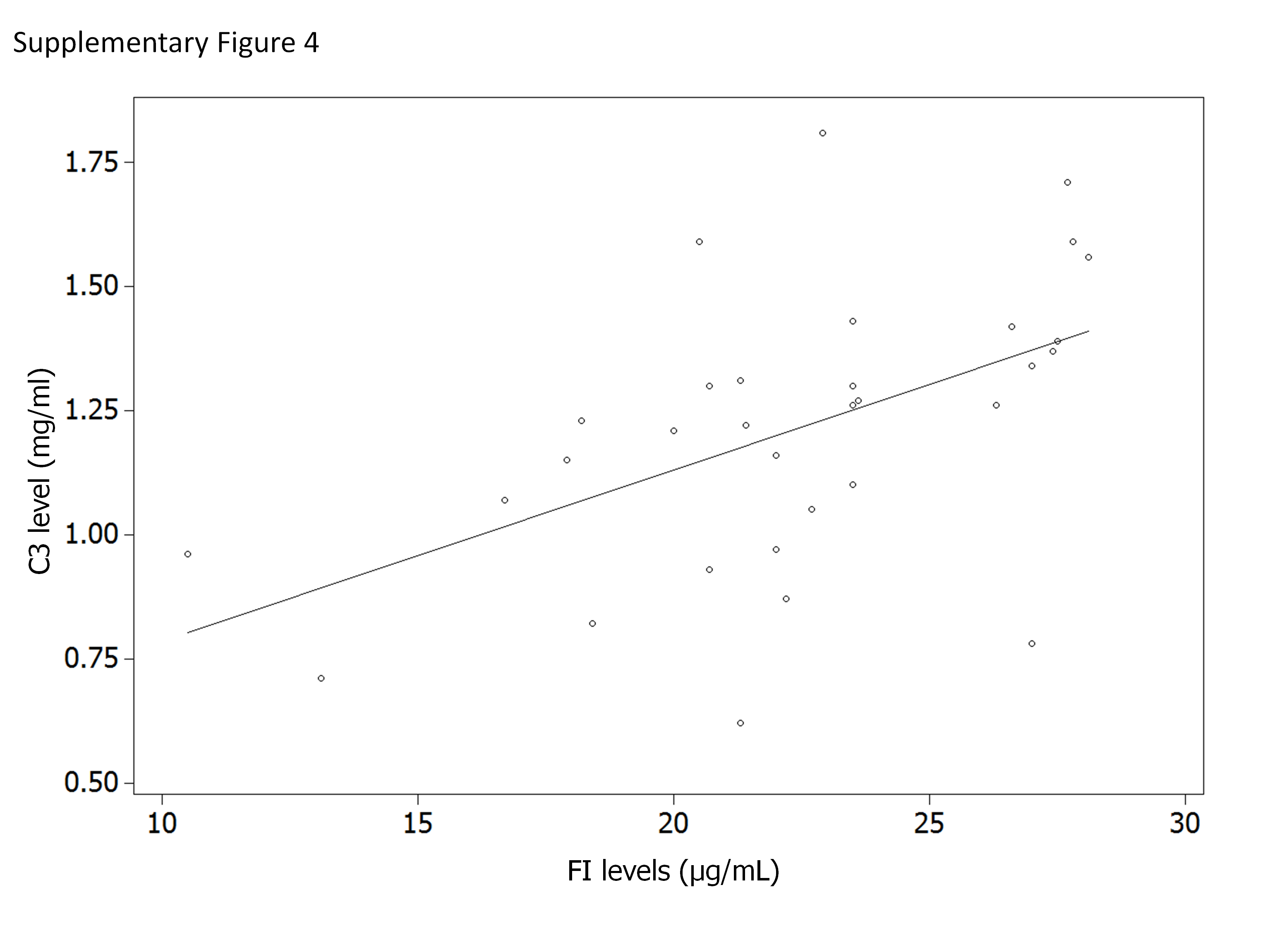
**

**Supplementary Figure 4.** Correlation between FI and C3 levels in individuals with AAMD and a type I *CFI* variant. Regression analysis was performed on the 32 patients with AAMD and a rare *CFI* variant associated with a low serum FI level. There was a statistically significant correlation between the FI level and C3 concentrations; (R = 0.51, P < 0.01).
