# Supplementary material for: Rare genetic variants in the CFI gene are associated with advanced age-related macular degeneration and commonly result in reduced serum factor I levels
Source: Hum Mol Genet. 2015 Mar 18;24(13):3861–70. doi: 10.1093/hmg/ddv091 (PMC4459386; doi:10.1093/hmg/ddv091)
Supplement: Supplementary Data [file supp_ddv091_ddv091supp_tables.doc]

**Supplementary Table 1: Rare *CFI* Coding Alleles I**n Sequencing Analysis

| SNP | Base Pair | Minor Allele | Opposite Allele | Total Affected | Total Unaffected | Amino Acid Change | Polyphen2 class |
| --- | --- | --- | --- | --- | --- | --- | --- |
| 4:110687980 | 110687980 | T | C | 0 | 1 | V20I | benign |
| 4:110687919 | 110687919 | C | T | 1 | 0 | H40R | benign |
| 4:110687908 | 110687908 | T | C | 2 | 0 | D44N | benign |
| rs144082872 | 110687890 | C | G | 2 | 0 | P50A | probably damaging |
| 4:110687878 | 110687878 | G | A | 1 | 0 | C54R | probably damaging |
| 4:110687876 | 110687876 | T | G | 0 | 1 | C54* | loss of function |
| 4:110687847 | 110687847 | A | G | 1 | 0 | P64L | probably damaging |
| 4:110687712 | 110687712 | G | T | 1 | 0 | E109A | benign |
| rs141853578 | 110685820 | T | C | 10 | 2 | G119R | probably damaging |
| 4:110685802 | 110685802 | T | C | 1 | 0 | G125R | probably damaging |
| 4:110685795 | 110685795 | G | A | 1 | 0 | V127A | possibly damaging |
| 4:110685721 | 110685721 | T | C | 2 | 0 | V152M | probably damaging |
| 4:110682846 | 110682846 | T | C | 4 | 0 | G162D | probably damaging |
| 4:110682801 | 110682801 | A | T | 1 | 0 | N177I | benign |
| rs200418129 | 110682781 | T | C | 0 | 1 | V184M | possibly damaging |
| rs143366614 | 110682771 | T | C | 1 | 0 | R187Q | possibly damaging |
| 4:110682739 | 110682739 | G | A | 1 | 0 | F198L | benign |
| rs149215929 | 110682726 | A | C | 5 | 7 | R202I | possibly damaging |
| rs138346388 | 110682723 | A | G | 2 | 0 | T203I | benign |
| 4:110682715 | 110682715 | T | A | 2 | 0 | Y206N | benign |
| 4:110682680 | 110682680 | G | C | 0 | 1 | Q217H | possibly damaging |
| 4:110681789 | 110681789 | T | G | 1 | 0 | S221Y | possibly damaging |
| rs190001845 | 110681781 | T | C | 1 | 0 | D224N | benign |
| 4:110681766 | 110681766 | G | A | 1 | 0 | C229R | probably damaging |
| 4:110681763 | 110681763 | T | C | 1 | 0 | V230M | probably damaging |
| rs146444258 | 110681732 | C | G | 12 | 1 | A240G | probably damaging |
| 4:110681708 | 110681708 | T | C | 1 | 0 | G248E | probably damaging |
| rs199688124 | 110681679 | T | C | 4 | 0 | A258T | possibly damaging |
| rs112534524 | 110681527 | T | C | 12 | 8 | G261D | benign |
| rs200544168 | 110681521 | A | C | 2 | 0 | G263V | probably damaging |
| 4:110681470 | 110681470 | T | C | 1 | 0 | G280D | possibly damaging |
| rs182078921 | 110681450 | T | C | 3 | 0 | G287R | probably damaging |
| rs11098044 | 110678925 | T | C | 5 | 4 | T300A | benign |
| 4:110673634 | 110673634 | T | G | 2 | 0 | D310E | possibly damaging |
| rs121964917 | 110670750 | A | G | 2 | 1 | R317W | benign |
| 4:110670749 | 110670749 | T | C | 1 | 0 | R317Q | benign |
| rs144164794 | 110670717 | T | C | 1 | 0 | G328R | probably damaging |
| 4:110670683 | 110670683 | T | C | 1 | 0 | R339Q | probably damaging |
| 4:110670680 | 110670680 | G | A | 1 | 0 | I340T | probably damaging |
| 4:110670459 | 110670459 | T | C | 0 | 1 | V355M | probably damaging |
| 4:110670456 | 110670456 | G | C | 1 | 0 | A356P | probably damaging |
| rs200619905 | 110670437 | G | C | 0 | 1 | G362A | benign |
| 4:110670416 | 110670416 | G | T | 1 | 0 | Y369S | probably damaging |
| 4:110670400 | 110670400 | A | C | 1 | 0 | W374C | probably damaging |
| 4:110667641 | 110667641 | T | C | 1 | 0 | R389H | benign |
| 4:110667612 | 110667612 | G | A | 1 | 0 | W399R | benign |
| rs139881195 | 110667600 | T | C | 1 | 0 | D403N | benign |
| rs74817407 | 110667590 | T | C | 1 | 6 | R406H | possibly damaging |
| rs61733901 | 110667561 | G | T | 1 | 0 | I416L | benign |
| rs121964912 | 110667554 | A | T | 2 | 0 | H418L | probably damaging |
| rs41278047 | 110667485 | C | T | 30 | 13 | K441R | benign |
| 4:110667431 | 110667431 | G | T | 1 | 0 | Y459S | possibly damaging |
| rs143827877 | 110667421 | A | T | 0 | 1 | Q462H | possibly damaging |
| 4:110667408 | 110667408 | G | A | 1 | 0 | C467R | probably damaging |
| rs121964913 | 110667387 | A | G | 2 | 0 | R474* | loss of function |
| 4:110667386 | 110667386 | T | C | 1 | 0 | R474Q | possibly damaging |
| 4:110667378 | 110667378 | G | C | 2 | 0 | D477H | possibly damaging |
| 4:110667377 | 110667377 | G | C | 4 | 0 | Splice site donor* | loss of function |
| 4:110663722 | 110663722 | A | C | 1 | 0 | G487C | probably damaging |
| rs200025458 | 110663707 | G | T | 1 | 0 | I492L | benign |
| 4:110663683 | 110663683 | T | C | 1 | 0 | G500R | benign |
| 4:110663677 | 110663677 | A | G | 1 | 0 | R502C | probably damaging |
| 4:110663647 | 110663647 | T | C | 1 | 0 | G512S | probably damaging |
| 4:110662193 | 110662193 | T | A | 0 | 1 | N536K | possibly damaging |
| 4:110662179 | 110662179 | T | C | 1 | 0 | W541* | loss of function |
| 4:110662178 | 110662178 | T | C | 1 | 0 | W541* | loss of function |
| 4:110662173 | 110662173 | G | A | 1 | 0 | V543A | possibly damaging |
| rs113460688 | 110662144 | A | G | 26 | 6 | P553S | benign |
| 4:110662140 | 110662140 | A | T | 3 | 1 | E554V | probably damaging |
| 4:110662068 | 110662068 | G | A | 2 | 0 | I578T | probably damaging |
| 4:110662063 | 110662063 | A | G | 1 | 0 | Q580* | loss of function |

*Loss of function variants

**Supplementary Table 2: Rare *CFI* Synonymous Alleles**

| SNP | Base Pair | Minor Allele | Opposite Allele | Total Affected | Total Unaffected | Amino Acid Change |
| --- | --- | --- | --- | --- | --- | --- |
| 4:110723116 | 110723116 | T | A | 1 | 0 | L4 |
| rs146462954 | 110687909 | A | G | 1 | 0 | C43 |
| 4:110687846 | 110687846 | T | C | 1 | 0 | P64 |
| 4:110687828 | 110687828 | G | A | 1 | 0 | N70 |
| 4:110687750 | 110687750 | C | T | 0 | 1 | P96 |
| 4:110685791 | 110685791 | C | T | 0 | 1 | E128 |
| 4:110682800 | 110682800 | G | A | 1 | 0 | N177 |
| 4:110682707 | 110682707 | G | A | 0 | 1 | D208 |
| rs140824635 | 110682686 | G | A | 0 | 2 | Y215 |
| 4:110681722 | 110681722 | T | A | 0 | 1 | G243 |
| 4:110670738 | 110670738 | G | A | 1 | 0 | L321 |
| rs141961923 | 110667460 | T | C | 0 | 1 | S449 |
| rs145296508 | 110662253 | G | A | 3 | 1 | G516 |

**Supplementary Table 3: Single Variant Analysis For** Association With Advanced AMD

| SNP | Base Pair | Minor Allele | Frequency in Affected | Frequency | Reference Allele | Total Affected | Total Unaffected | P-value | Odds Ratio | Amino Acid Change |
| --- | --- | --- | --- | --- | --- | --- | --- | --- | --- | --- |
|  |  |  |  | in Unaffected |  |  |  |  |  |  |
| rs141853578 | 110685820 | T | 0.002207 | 0.000714 | C | 10 | 2 | 0.15 | 3.09(0.66-29.08) | G119R |
| rs149215929 | 110682726 | A | 0.001197 | 0.003418 | C | 5 | 7 | 0.070 | 0.35(0.09-1.28) | R202I |
| rs146444258 | 110681732 | C | 0.002648 | 0.000357 | G | 12 | 1 | 0.023 | 7.43(1.10-317.46) | A240G |
| rs112534524 | 110681527 | T | 0.002649 | 0.002859 | C | 12 | 8 | 1.00 | 0.93(0.35-2.62) | G261D |
| rs11098044 | 110678925 | T | 0.001104 | 0.00143 | C | 5 | 4 | 0.74 | 0.77(0.17-3.89) | T300A |
| rs74817407 | 110667590 | T | 0.000221 | 0.002143 | C | 1 | 6 | 0.015 | 0.10(0.002-0.85) | R406H |
| rs41278047 | 110667485 | C | 0.006623 | 0.004643 | T | 30 | 13 | 0.35 | 1.43(0.72-2.99) | K441R |
| rs113460688 | 110662144 | A | 0.005737 | 0.002143 | G | 26 | 6 | 0.027 | 2.69(1.08-7.99) | P553S |

Variants with counts in cases and controls ≥ 5 are analyzed individually for significance.

**Supplementary Table 4: Pooled Analysis For Variants With Counts < 5**

|  | Total Affected | Total Unaffected | P-value |
| --- | --- | --- | --- |
|  |
| Benign | 20 | 3 | 0.029 |
| Possibly Damaging | 15 | 4 | 0.16 |
| Probably Damaging | 36 | 2 | 5.0 x 10^-6^ |
| Loss of Function | 9 | 1 | 0.10 |
|  |  |  |  |
| Loss of Function or Probably Damaging | 45 | 3 | 6.1 x 10^-7^ |
| All non-synonymous variants | 80 | 10 | 1.1 x 10^-8^ |
| Synonymous variants | 9 | 8 | 0.47 |
